# Mitigating Diseases Transmitted by *Aedes* Mosquitoes: A Cluster-Randomised Trial of Permethrin-Impregnated School Uniforms

**DOI:** 10.1371/journal.pntd.0005197

**Published:** 2017-01-19

**Authors:** Pattamaporn Kittayapong, Phanthip Olanratmanee, Pongsri Maskhao, Peter Byass, James Logan, Yesim Tozan, Valérie Louis, Duane J. Gubler, Annelies Wilder-Smith

**Affiliations:** 1 Center of Excellence for Vectors and Vector-Borne Diseases, Faculty of Science, Mahidol University at Salaya, Nakhon Phatom, Thailand; 2 Department of Biology, Faculty of Science, Mahidol University, Bangkok, Thailand; 3 Faculty of Science and Technology, Rajabhat Rajanagarindra University, Chachoengsao, Thailand; 4 Faculty of Humanities and Social Sciences, Rajabhat Rajanagarindra University, Chachoengsao, Thailand; 5 Umeå Centre for Global Health Research, Epidemiology and Global Health, Department of Public Health and Clinical Medicine, Umeå University, Umeå, Sweden; 6 London School of Hygiene and Tropical Medicine, London, United Kingdom; 7 Institute of Public Health, Heidelberg University Medical School, Germany; 8 College of Global Public Health, New York University, New York, United States; 9 Emerging Infectious Diseases Program, Duke-NUS Graduate Medical School, Singapore; 10 Lee Kong Chian School of Medicine, Nanyang Technological University, Singapore; 11 Institute of Public Health, University of Heidelberg, Heidelberg, Germany; North Carolina State University, UNITED STATES

## Abstract

**Background:**

Viral diseases transmitted via *Aedes* mosquitoes are on the rise, such as Zika, dengue, and chikungunya. Novel tools to mitigate Aedes mosquitoes-transmitted diseases are urgently needed. We tested whether commercially insecticide-impregnated school uniforms can reduce dengue incidence in school children.

**Methods:**

We designed a cluster-randomised controlled trial in Thailand. The primary endpoint was laboratory-confirmed dengue infections. Secondary endpoints were school absenteeism; and impregnated uniforms’ 1-hour knock-down and 24 hour mosquito mortality as measured by standardised WHOPES bioassay cone tests at baseline and after repeated washing. Furthermore, entomological assessments inside classrooms and in outside areas of schools were conducted.

**Results:**

We enrolled 1,811 pupils aged 6–17 from 5 intervention and 5 control schools. Paired serum samples were obtained from 1,655 pupils. In the control schools, 24/641 (3.7%) and in the intervention schools 33/1,014 (3.3%) students had evidence of new dengue infections during one school term (5 months). There was no significant difference in proportions of students having incident dengue infections between the intervention and control schools, with adjustment for clustering by school. WHOPES cone tests showed a 100% knock down and mortality of *Aedes aegypti* mosquitoes exposed to impregnated clothing at baseline and up to 4 washes, but this efficacy rapidly declined to below 20% after 20 washes, corresponding to a weekly reduction in knock-down and mosquito mortality by 4.7% and 4.4% respectively. Results of the entomological assessments showed that the mean number of *Aedes aegypti* mosquitoes caught inside the classrooms of the intervention schools was significantly reduced in the month following the introduction of the impregnated uniforms, compared to those collected in classrooms of the control schools (p = 0.04)

**Conclusions:**

Entomological assessments showed that the intervention had some impact on the number of *Aedes* mosquitoes inside treatment schools immediately after impregnation and before insecticidal activity declined. However, there was no serological evidence of protection against dengue infections over the five months school term, best explained by the rapid washing-out of permethrin after 4 washes. If rapid washing-out of permethrin could be overcome by novel technological approaches, insecticide-treated clothes might become a potentially cost-effective and scalable intervention to protect against diseases transmitted by *Aedes* mosquitoes such as dengue, Zika, and chikungunya.

**Trial Registration:**

ClinicalTrials.gov NCT01563640

## Author Summary

Viral diseases transmitted via *Aedes* mosquitoes are on the rise, such as Zika, dengue, and chikungunya. Novel tools to mitigate Aedes mosquitoes-transmitted diseases are urgently needed. We tested whether commercially available insecticide-impregnated school uniforms can reduce dengue incidence in school children. To test this hypothesis we designed a school based randomized controlled trial where we enrolled 1,811 school children aged 6–17. For study monitoring, we also measured the effect of the impregnated uniforms on the survival of *Aedes* mosquitoes based on a standard bioassay test called WHOPES cone test. Furthermore, we counted the number of *Aedes* mosquitoes in classrooms and outside areas of classrooms. In the control schools, 3.7% and in the intervention schools 3.3% of the students had evidence of new dengue infections during the 5 month long school term, which indicates that there was no protection against dengue infections despite the fact that the knockdown effect of the impregnated uniforms was very high in the laboratory. We also showed a significant reduction of Aedes mosquitoes in the classrooms of the intervention schools. So why did this not translate into clinical protection against dengue? We assume the reason was the rapid wash-out effect of permethrin. Despite the company’s claim that impregnated clothing would withstand up to 70 launderings, we found a rapid decline in permethrin efficacy already after 4 washes, with the efficacy to below 20% after 20 washes. If rapid washing-out of permethrin could be overcome by novel technological approaches, insecticide-treated clothes might become a potentially cost-effective and scalable intervention to protect against diseases transmitted by *Aedes* mosquitoes such as dengue, Zika, and chikungunya.

## Introduction

*Aedes* is a genus of mosquitoes originally found in tropical and subtropical zones, but which is now widespread in most continents. The current geographical distribution is the widest ever recorded and there is potential for further spread [1]. Over 50% of the world’s population live in areas where they are at risk of *Aedes* transmitted infections [2]. *Aedes* mosquitoes transmit a large array of viral diseases, notably dengue, Zika, yellow fever, West Nile encephalitis, chikungunya viruses. *Aedes* mosquitoes are predominantly active during daylight hours, well adapted to urban living, and are difficult to control with currently available control strategies [3]. Although novel approaches, such as Wolbachia-infected mosquitoes or genetically modified male mosquitoes, appear promising [4,5], their use remains controversial and they are unlikely to be implemented at a large scale in the near future. The current Zika virus outbreak in Latin America, associated with severe complications such as microcephaly and neurological complications [6], accentuates the urgent need to develop additional novel approaches that are simple and rapidly scalable; in particular personal protection approaches to protect individuals at high risk such as pregnant women. Since *Aedes aegypti* is a day-biting mosquito, developing technologies that can be applied during the day to offer protection against mosquito bites should be top priority.

Permethrin is a pyrethroid-based insecticide registered by the US Environmental Protection Agency (EPA) since 1977 that has been extensively used as insect repellent and insecticide with a documented safety record [7]. Permethrin can be bound to fabric fibres in clothing via different techniques such as micro-encapsulation and polymer coating [8,9]. Insecticide-treated clothing has been used for many years by the military and in recreational activities as personal protection against bites from a variety of arthropods including ticks, chigger mites, sandflies and mosquitoes and is thought to be safe [7]. Insecticide-treated clothing has been reported to give between 0% and 75% protection against malaria and between 0% and 79% protection against leishmaniasis [7]. No field trials have been conducted to demonstrate the efficacy against Aedes-transmitted diseases. However, one study showed that wearing permethrin-treated clothing resulted in a reduction in the number of Aedes bites by 90% [10] suggesting that it could potentially be a promising intervention for *Aedes*-transmitted diseases such as Zika, dengue, and chikungunya.

Of all *Aedes*-transmitted diseases, dengue is the most frequent arboviral disease globally, with an estimated 390 million infections annually [11]. Children carry a significant burden of morbidity and mortality for dengue with a higher rate of more severe disease than adults [12]. Because children spend most of their day at school at a time of peak biting behaviour of *Aedes* mosquitoes, personal protective measures such as impregnated clothing should be investigated. As school children in most dengue endemic countries wear school uniforms as a social norm, impregnated school-uniforms could be easily scaled up as a national programme, if such a strategy were proven to be impactful.

InsectShield manufactures permethrin-impregnated apparel for recreational and military purposes. InsectShield Repellent Apparel is registered by the US EPA [13]; their approach is a polymer-coating technique which is claimed to withstand up to 70 washings [13]. InsectShield clothing was successfully used for tick bite prevention [14]and also protective against mosquito bites by measuring changes in antibody titers to mosquito salivary gland extracts [15]. We conducted a school based field trial to assess the efficacy of InsectShield school uniforms on reducing dengue infections in children.

## Methods

We conducted a randomised controlled trial blinded over a school-term (5 months) in Thailand to evaluate the effectiveness of insecticide-treated school uniforms for the prevention of dengue in school children under field conditions. The trial was funded by the European Commission under the 7^th^ framework (Grant No. 282589) and registered at www.clinicaltrial.gov: NCT01563640).

### Setting and participants

The study area is located about 150 km east of Bangkok, in Plaeng Yao District, Chachoengsao Province, Eastern Thailand. The region covers an area of 237 km^2^ with a population of 36,607, with a total of 24 schools. Official permission from the Office of Basic Education Commission was obtained. School directors were informed about the trial, and 10 school directors agreed to participate. In these 10 schools, students in grades one to nine, aged 6 to 17 years, were eligible to participate if parental consent was given. Awareness seminars for the school senior management, teachers and parents were held prior to the trial to ensure high recruitment rates and compliance. The rainy season associated with the highest dengue incidence runs from June to October, and the school term (corresponding to the study period) was from mid-June to mid-November.

The protocol of this school-based trial was reviewed and approved by the Mahidol University Institutional Review Board (MU-IRB 2009/357.1512).

### Intervention

The intervention was permethrin-impregnated school uniforms. The impregnation process involved washing and then coating the uniforms in a proprietary process resulting in 0.054 mg/m^2^ permethrin (InsectShield USEPA2009). As, hypothetically, individuals wearing insecticide-impregnated clothing could also provide indirect protection to others not wearing impregnated clothing, we randomised the intervention by school, rather than by individual. To ensure acceptability by the school senior management and to ensure real-life scenarios, we used locally used school uniforms, comprising the standard school uniform, Scouts uniform, sports uniform and cultural uniform. Uniforms were typically short-sleeved and only covered the legs down to the knees (shorts or skirts).

### Randomisation

Computer randomisation into intervention versus control group was by school, randomised into equal groups. Only the investigator who carried out the randomisation in Sweden and the overseas impregnating factory (InsectShield) were aware of the allocation; schools, children and on-site investigators were blinded. To maintain blinding for the on-site investigators and schools, all uniforms (from both intervention and control schools) were collected and sent to the InsectShield impregnating factory, but only the uniforms of the intervention schools were impregnated. To ensure that the correct uniforms were returned to the correct owners, all uniforms received labels indicating the child’s name, class, and school.

### Paired blood samples

We collected blood samples via finger-prick (<0.2ml) from study subjects at the beginning and end of the school term (June and November). Dengue IgG ELISA was first performed for all paired samples. A primary infection was defined as a seroconversion from baseline negative IgG to positive IgG at follow-up. If the baseline sample was IgG positive, we did an additional analysis to identify new dengue infections by using the monoclonal antibody (MAb)-based capture enzyme-linked immunosorbent assays (MAb-ELISA) to measure the increase in IgG, whereby Dengue IgG indirect ELISA was performed using purified 2H2 monoclonal antibody for coating plates on paired samples and cut-off values used as described by Johnson et al [16].

### Efficacy of permethrin-impregnated school uniforms against *Aedes* mosquitoes at baseline and after washing

Impregnated standard uniforms were evaluated for their effect on mosquito knock-down and 24-hour mortality at baseline and after repeated washing, measured by standardised WHOPES bioassay cone tests [17]. A WHO plastic cone was secured onto the cloth using rubber bands. Batches of five nulliparous starved female *Aedes aegypti* mosquitoes (3–5 days old) were placed in the cone via a mouth aspirator, and a small cotton plug was used to close the aperture. Bioassays were carried out at 25 ± 2°C and 65 ± 10% relative humidity. Mosquitoes were exposed to the materials for three minutes and removed using a mouth aspirator. The mosquitoes were then placed in a holding cup inside an insectary (25 ± 2°C and 65 ± 10%) with a net secured over the top with two elastic bands and cotton wool soaked in 10% sugar solution. Knock-down was recorded one hour post exposure, and mortality was recorded after 24 hours. Ten replicates were carried out for each sample of 10 shirts and 10 skirts or trousers. Testing was done at baseline and repeated after weekly laundering where the uniforms were hand-washed and dried in the shade for 24 hours to simulate field conditions.

### Entomological assessments in classrooms and school corridors

Portable vacuum aspirators were used to collect adult mosquitoes indoors in the school areas at baseline and monthly following the introduction of treated uniforms. The collectors aspirated mosquitoes in 5 classrooms per school and spent 15 minutes per classroom for aspiration. BG sentinel traps, one trap per school, were placed in the corner of the corridor of each school building outside the classrooms. They were left in both treatment and control schools in the morning and were collected in the evening of the same day. The collectors collected mosquitoes from these traps in all schools on two consecutive days.

Mosquitoes collected were transferred to plastic tubes and were stored on ice during the transport to the laboratory at the Center of Excellence for Vectors and Vector-Borne Diseases in Salaya Campus of Mahidol University for further processing. The collected mosquitoes were then sorted, counted and identified to genus and species according to each school before storage at -80°C.

### Absenteeism

School class teachers recorded all children who were absent for at least one day. Children or their parents were contacted by phone to obtain the reason of absenteeism. Absenteeism for sick leave (for any cause, with or without fever) for at least one day was recorded, and those who were absent from school for at least 2 days due to a febrile illness were also documented.

### Outcomes

The primary outcome was the incidence of laboratory confirmed dengue infections during the school term in individuals wearing impregnated uniforms versus non-impregnated uniforms. Secondary outcomes were (1) the number of *Aedes aegypti* mosquitoes in intervention and control schools (in classrooms, and school corridors) and (2) the 1 hour knock-down and 24 hour mortality of *Aedes aegypti* mosquitoes exposed to our impregnated school uniforms as measured by WHOPES cone tests at baseline and after weekly washing. We also recorded school absenteeism for 1 day or more, and also absenteeism for 2 days or more because of a febrile illness.

### Sample size calculation and statistical analysis

The sample size calculation based on the primary endpoint has been described in detail in the trial protocol [18]. We originally planned a cross-over design spanning two transmission seasons because dengue transmission can vary greatly between schools and seasons, which can to some extent be controlled for by a cross-over design. The original assumptions underlying the sample size for the trial were an incidence rate (symptomatic plus asymptomatic) averaging 5% during a transmission season [19], i.e. 10% over two seasons; an effect size of halving incidence by using impregnated uniforms, and a drop-out rate (children leaving the school or withdrawing from the trial, etc.) of 20%. Taking into account a conservative cluster design effect of 3, the total sample size was calculated to be 2,012 (i.e. 1,006 in each study arm). Retaining the design effect of 3 (since we did not gain appreciable understanding of the local geographic and temporal variability in dengue incidence), a sample size of 270 x 3 in each arm of the curtailed trial would have given 80% power to detect a significant difference of 7% versus 2% incidence between the two groups (alpha = 0.05). The total documented enrolment of 1,655 approximated to this sample size of 270 x 3 x 2 = 1,620. The 7% versus 2% difference corresponds to our original design assumption that a difference of at least 5% would be necessary in order to have policy implications.

Statistical analyses used STATA 12. The effectiveness of the intervention was determined by comparing proportions of students in intervention and control schools who had confirmed incident dengue infections during the trial, with adjustment for clustering by school as the unit of randomisation, using the **clchi2** command. Differences in mean monthly numbers of *Aedes* mosquitoes trapped at intervention and control schools during the month in which the intervention was efficacious were assessed using the **ttest** command on ln(n+1) transformed values.

## Results

### Participant flow and baseline data

**Fig 1:**
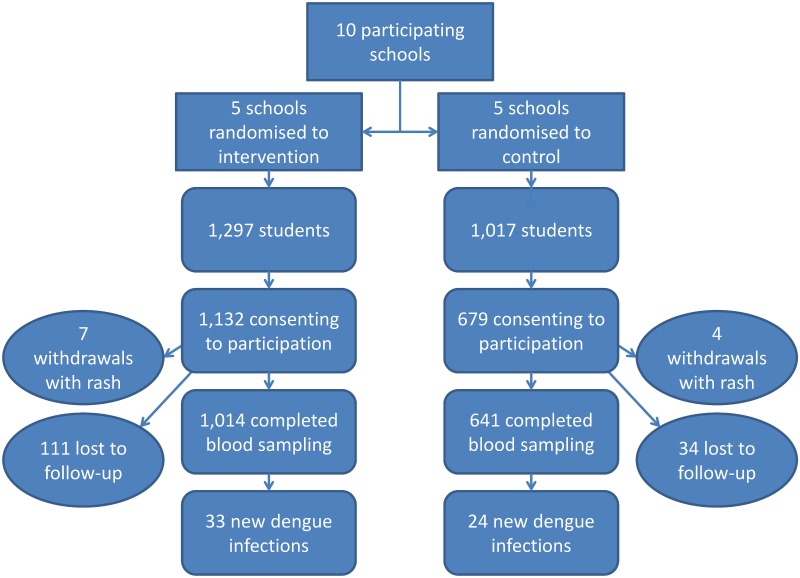
Flowchart showing numbers of participants in both intervention and control groups at all stages of the trial.

The flow chart (Fig 1) summarizes the numbers of participants in both intervention and control groups at all stages of the trial. The ten participating schools had 2,314 students at the start of the trial; consent was given by 1,811 students’ parents. Out of the 1,811 enrolled students (mean age 10.1 years; range 6–17; 908 males, 903 females), 11 were withdrawn because of skin irritation, which were all mild and transient (7 in intervention; 4 in control schools). Of the 1,800, 1,655 provided complete sets of paired blood samples (Table 1). The IgG baseline dengue seropositivity rate was 53.0% (878/1,655).

**Table 1:** Proportions of dengue infections in 1,655 Thai school children with paired serum samples at 10 schools randomised to insecticide-treated school uniforms (intervention) or untreated school uniforms (control).

		Participation					Dengue results				
School	Group	Number of eligible students	Number of study participants	% participation	Median age	% females	Number of paired blood tests	Baseline positive dengue IgG	% baseline positive	Number of laboratory confirmed dengue infections during study period	% lab-confirmed dengue infections during study period
A	control	240	170	70.8	11.59	54.7	152	91	59.9	15	9.9
B		370	294	79.5	9.14	51.7	285	150	52.6	3	1.1
C		169	75	44.4	9.73	38.7	68	31	45.6	6	8.8
D		44	28	63.6	9.50	46.4	26	8	30.8	0	0
E		194	112	57.7	9.57	51.8	110	59	53.6	0	0
All controls		1017	679	66.8	9.73	50.8	641	339	52.9	24	3.7
F	intervention	345	325	94.2	10.81	48.6	262	125	47.7	5	1.9
G		214	170	79.4	9.50	44.7	167	70	41.9	8	4.8
H		274	234	85.4	10.76	53.0	224	137	61.2	9	4.0
I		416	358	86.1	10.55	49.2	346	203	58.7	11	3.2
J		48	45	93.8	8.79	53.3	15	4	26.7	0	0
All interventions		1297	1132	87.3	10.51	49.3	1014	539	53.2	33	3.3
Overall		2314	1811	78.3	10.13	49.9	1655	878	53.1	57	3.4

### Outcomes

Of the 1,655 students with paired samples, 57 had evidence for a dengue infection during the study period, and 16 had equivocal results. Between schools, the number of dengue infections varied considerably from 0 to 9.9% (Table 1). In the control schools, 24/641 (3.7%) and in the intervention schools 33/1,014 (3.3%) students had evidence of new dengue infection. For our primary outcome, there was no significant difference in proportions of students having incident dengue infections between the intervention and control schools, with adjustment for clustering by school (χ^2^ = 0.02, p = 0.89).

The proportion of absenteeism (for any reason) was relatively high in all schools and ranged from an average of 23.0% (SD = 15.9%) over the school term, with a range of 9.6% to 49.9% in the treatment schools and 0.9% to 32.0% in the control schools. The proportion of pupils not going to school for at least 2 days due to a febrile illness ranged from an average of 1.4% (SD = 1.17%) over the school term from 0.6.% to 4.0% in the treatment schools and from 0.1% to 2.8% in the control schools. The overall proportion of absenteeism due to fever of at least 2 days was relatively stable over the school term. There were no statistical differences between the treatment and control groups for any of the school months.

**Fig 2:**
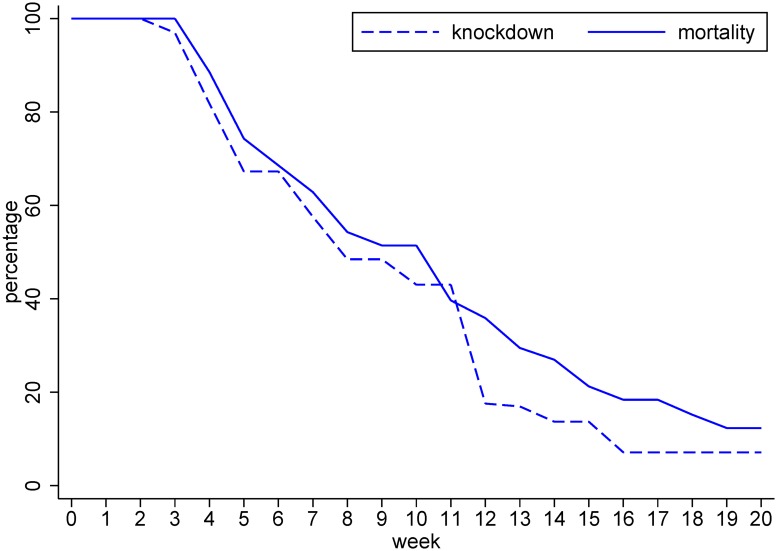
The effect of repeated washing on knock-down (after 1 hour) and mortality (after 24 hours) of *Aedes aegypti* after being exposed to permethrin-impregnated school uniforms.

Fig 2 shows both 1 h knock-down and 24 h mortality of *Aedes* mosquitoes exposed to the impregnated school uniforms. This started close to 100% and remained at high levels for up to 4 washes. After 4 washes, both knock-down and mortality declined rapidly. After 20 washes, the efficacy was below 20%. Knock-down decreased at an approximately linear rate of 4.7% per week, mortality at 4.4% per week. Due to this unexpectedly rapid waning of intervention efficacy, although the study was originally planned as a cross-over trial covering two dengue transmission seasons, we decided to abandon the second phase of the cross-over trial design.

Results of the entomological assessments showed that the mean number of *Aedes aegypti* mosquitoes caught inside the classrooms of the intervention schools was significantly lower in the month following the introduction of the impregnated uniforms, when compared to those collected in classrooms of the control schools (back-transformed mean of ln(n+1) transform in control schools 4.9, in intervention schools 1.4; ratio = 3.5, 95% CI 1.1 to 5.5). There was no significant difference in the mean numbers of *Aedes aegypti* mosquitoes collected at other times (control 4.0, intervention 3.8; ratio = 1.1, 95% CI 0.7 to 1.7).

## Discussion

Given the day-biting behaviour of *Aedes* mosquitoes, impregnated school uniforms could potentially be a simple novel tool to reduce mosquito-borne diseases and local vector populations. Our results from the WHOPES cone tests at the start of the trial underpin the potential for insecticide-treated uniforms to protect against dengue by reducing the populations of *Aedes* mosquitoes and hence mosquito-bites: knock-down effect and mortality immediately after impregnation of uniforms with permethrin by the InsectShield proprietary method were close to 100%, consistent with results obtained under laboratory conditions at the London School of Hygiene and Tropical Medicine. [20] Furthermore, we documented a significant reduction in *Aedes* mosquito numbers in the classrooms of the intervention schools in the first month after the start of the trial at the time when the insecticidal activity of impregnated uniforms was still 100%.

However, our cluster- randomised controlled trial in ten Thai schools involving 1,811 children did not show serological evidence of a protective effect over the 5-month study period of one school term. Given the theoretical support for this strategy, we need to carefully examine plausible reasons for the apparent failure to protect in our trial, so that this intervention is not discarded as an ineffective strategy for control against *Aedes*-transmitted diseases such as dengue, Zika or chikungunya. The main reason for the negative result was the rapidly waning efficacy of InsectShield permethrin-impregnated clothes under field conditions. We chose InsectShield factory-impregnation over hand-dipping with permethrin because InsectShield impregnation (unlike hand-dipping with permethrin) results in odourless, well tolerated apparel—a fact that is important for a double-blind randomised trial where odour could otherwise had given away the allocation group. We had not anticipated rapid washing-out of permethrin prior to the study as InsectShield have consistently claimed that the insecticidal efficacy of their proprietary method of permethrin impregnation withstands up to 70 washes [13]. However, we documented rapid declines in insecticidal activity after the first 4 washes. After 20 washes, the knock-down and mortality effects on mosquitoes were well below 20%. What might be the reasons for such rapid waning of insecticidal activity? Maybe the quality of already used cotton Thai school uniforms was inferior to clothing materials used by InsectShield for commercial purposes. With suboptimal cloth quality, the coating technique might have been less durable? Or maybe the washing conditions of the tropics, drying in the open air and ironing decreased the insecticidal effect more rapidly? However, we believe it was not just the potentially suboptimal cloth material of local Thai school uniforms, as a very recent study on laundering resistance of five commercially available, factory-treated permethrin-impregnated fabrics also found that permethrin content fell by 58.1 to 98.5% after 100 defined machine launderings, with InsectShield showing the fastest loss [21]. There is an urgent need for a standardised testing and licensing procedure for insecticide-impregnated commercial clothing to avoid misleading information.

We also documented a high heterogeneity in incident infections between the schools and differences in baseline seroprevalence, which may have masked the extent of the efficacy in our trial. Large differences in dengue infection rates between schools within the same year have also been noted by other groups [22,23], and underpin the difficulty in sample size calculations and subject selection of a disease that appears to be not only highly focal but also often exhibits a cyclical pattern with high and low epidemic years.

We need to consider other potential causes for the lack of efficacy in our trial. Although acceptability and compliance with the trial uniforms was high, [24] school uniforms are not worn after school and over weekends. We did some simulation modelling and estimated a reduction of dengue infections by 47% if 60% of all mosquito bites occurred during school hours and 70% of the children wore treated uniforms, assuming that mosquito knock-down and mortality levels remained at baseline (without washing-out effect) [25]. A reduction of dengue infections by 47% would indeed be a major public health victory.

Because we used paired blood samples at baseline and at the end of the study period (5 months), we are unable to tease out the impact of impregnated clothing on dengue incidence in the first month of wearing the intervention uniforms, at a time when the efficacy in terms of knock-down and mortality effect on mosquitoes was still close to 100%. As a proxy marker for symptomatic dengue disease, we recorded absenteeism of 2 days due to a febrile illness, but found no differences between the treatment and control groups, nor did we find statistically significant differences from month to month. Nevertheless, the results of entomological assessments showed that the intervention had an impact on the number of *Aedes* mosquitoes inside intervention schools in the first month of the intervention before insecticidal activity declined. This is an important finding that encourages continued research on the use of insecticide-treated clothing as a potential strategy for dengue prevention in school children.

Permethrin-treated clothes were also shown to be effective in reducing tick bites and mosquito bites in general in other studies, however, similarly as in our study, this effect waned off as time passed on, possibly also due to laundering effect [14,15]. Long-lasting insecticide-treated bednets were a major breakthrough in the control of malaria. However, bednets do not get washed so frequently. For insecticide-treated clothing to be a viable public health intervention it should withstand regular washing. If the rapid washing-out of permethrin can be overcome by novel technological approaches, insecticide-treated clothes would deserve to be re-evaluated as a potentially cost-effective and scalable intervention. Despite the urgency of the current Zika outbreak associated with serious pregnancy outcomes, we should not rush into recommending factory-impregnated clothing to pregnant women in Zika affected areas, until standardised testing and licensing procedures for insecticide-treated materials are implemented, with defined cut-off values for initial maximum and post-laundering minimum concentrations of permethrin as well as data on toxicity, homogeneity on fabrics, residual activity, and laundering resistance [21]. Failing to do this could generate a dangerous sense of security among Zika-exposed pregnant women using impregnated clothing, since the wearer has no means of judging insecticidal efficacy. Given the increasingly epidemic proportions of *Aedes*-transmitted viral infections, we hope that the findings from this trial will provide strong impetus to fund research to develop appropriate and safe technologies for long-lasting insecticide-treated clothing materials that can be used for school uniforms, work place uniforms and maternity clothing alike.

## Supporting Information

S1 CONSORT 2010 Checklist (DOCX)

S1 Trial Protocol (PDF)
